# Supplementary material for: Examining public acceptance of AI versus human-centric dementia care across NHS England’s dementia pathway stages
Source: PLOS Digit Health. 2026 Oct 8;5(10):e0001088. doi: 10.1371/journal.pdig.0001088 (PMC13648964; doi:10.1371/journal.pdig.0001088)
Supplement: Supplementary file 1 [file pdig.0001088.s001.docx]

Examining public acceptance of AI versus human-centric dementia care across NHS England’s dementia pathway stages

Thomas O’Fee ^1, 2, 3^ *, Santosh Vijaykumar ^1, 2, 3^ , Michael Craig ^1, 3^

^1^ Climate & Health Infodemic Resilience Programme (CHIRP), Northumbria University, Newcastle upon Tyne, United Kingdom

^2^ Psychology and Communication Technology (PaCT) Lab, School of Psychology, Faculty of Health and Wellbeing, Northumbria University, Newcastle upon Tyne, United Kingdom

^3^ School of Psychology, Faculty of Health and Wellbeing, Northumbria University, Newcastle upon Tyne, United Kingdom

* Corresponding author

Email: [thomas.ofee@northumbria.ac.uk](mailto:thomas.ofee@northumbria.ac.uk) (TO)

**Supplementary Covariate Results**

**Influence of Dementia Knowledge and AI Literacy**

Analyses for the acceptability and perceived likelihood measures were replicated using scores from the Artificial Intelligence Literacy Scale (AILS), Dementia Attitude Scale (DAS), and Dementia Knowledge Assessment Tool (DKAT) as covariates.

**Overall Effects.** Introducing DKAT, DAS and AILS scores as covariates did not change the overall results from the primary analysis. For acceptability, the three-way interaction was upheld (*F*(4, 1497) = 39.21, *p* < .001). As were the main effects of stage (*F*(4, 1497) = 19.70, *p* <.001) and involvement (*F*(1, 1497) = 149.29, *p* < .001) , and all established two-way interaction effects. For likelihood, main effects were all upheld; stage (*F*(4, 1497) = 2.56, *p* = .037), involvement (*F*(1, 1497) = 415.64, *p* < .001), centrism (*F*(1, 1497) = 51.29, *p* < .001). All two-way interactions were also upheld; however, the three-way interaction was diminished (*F*(4, 1497) = 1.62, *p* = .166).

**Preventing-well stage.** At the preventing-well stage, the introduction of the covariates made no significant difference to the previously established interaction between involvement and centrism on acceptability (*F*(1, 297) = 44.81, *p* <.001). For likelihood, the established main effect of involvement was also upheld (*F*(1, 297) = 87.30, *p* < .001).

**Diagnosing-well stage.** At the Diagnosing-well stage, the significant interaction effect between involvement and centrism was upheld (*F*(1, 255) = 95.46, *p* <.001). Similarly, for likelihood, the main effects of involvement and centrism were upheld (*F*(1, 255) = 118.68, *p* <.001; *F*(1, 255) = 10.05, *p* = .002) as well as the significant interaction between involvement and centrism (*F*(1, 255) = 5.59, *p* = .023).

**Supporting-well stage.** All significant effects from the primary analysis were upheld when introducing he covariates. Including the main effects of involvement (*F*(1, 297) = 77.24, *p* < .001) and centrism (*F*(1, 7297 = 5.04, *p* < .05) as well as the 2-way interaction between involvement and centrism (*F*(1, 297) = 244.92, *p* < .001) for acceptability. For likelihood, main effects were upheld for involvement (*F*(1, 297) = 72.60, *p* < .001) and for centrism (*F*(1, 297) = 7.12, *p* = .008).

**Living-well stage.** Similarly, the covariate analysis at the living-well stage revealed no significant changes to the established main effect of involvement (*F*(1, 297) = 144.74, *p* < .001), and the interaction between involvement and centrism (*F*(1, 297) = 103.06, *p* < .001) on acceptability. The main effects of involvement and centrism on ratings of likelihood were also upheld (*F*(1, 297) = 90.65, *p* < .001; *F*(1, 297) = 24.87 = *p* < .001).

**Dying-well stage.** Finally, all previously established effects at the dying-well stage were also upheld with the covariates. For acceptability, there was a significant main effect of involvement (*F*(1, 297) = 34.57, *p* <.001) and a significant interaction effect between involvement and centrism (*F*(1, 297) = 546.13, *p* <.001). For likelihood, there were significant main effects for involvement (*F*(1, 297) = 58.42, *p* < .001) and for centrism (*F*(1, 297) = 30.51, *p* < .001)
